# Continuous behavioural ‘switching’ in human spermatozoa and its regulation by Ca^2+^-mobilising stimuli

**DOI:** 10.1093/molehr/gaz034

**Published:** 2019-06-13

**Authors:** Cosmas Achikanu, Joao Correia, Héctor A Guidobaldi, Laura C Giojalas, Christopher L R Barratt, Sarah Martins Da Silva, Stephen Publicover

**Affiliations:** 1 School of Biosciences, University of Birmingham, Birmingham, UK; 2 Universidad Nacional de Córdoba. Facultad de Ciencias Exactas, Físicas y Naturales, Centro de Biología Celular y Molecular, Av. Vélez Sarsfield 1611, CP X5016GCA, Córdoba, Argentina; 3 Instituto de Investigaciones Biológicas y Tecnológicas, UNC, CONICET, FCEFyN, Av. Vélez Sarsfield 1611, CP X5016GCA, Córdoba, Argentina; 4 Reproductive and Developmental Biology, School of Medicine, Ninewells Hospital and Medical School, Dundee DD19SY, UK; 5 University of Dundee, Dundee DD19SY, UK 4Assisted Conception Unit, Ninewells Hospital Dundee, Dundee DD19SY, UK; 6 Centre for Human Reproductive Science, University of Birmingham, UK

**Keywords:** spermatozoa, behaviour, motility, calcium, pH

## Abstract

Human sperm show a variety of different behaviours (types of motility) that have different functional roles. Previous reports suggest that sperm may reversibly switch between these behaviours. We have recorded and analysed the behaviour of individual human sperm (180 cells in total), each cell monitored continuously for 3–3.5 min either under control conditions or in the presence of Ca^2+^-mobilising stimuli. Switching between different behaviours was assessed visually (1 s bins using four behaviour categories), and was verified by fractal dimension analysis of sperm head tracks. In the absence of stimuli, ~90% of cells showed at least one behavioural transition (mean rate under control conditions = 6.4 ± 0.8 transitions.min^−1^). Type 1 behaviour (progressive, activated-like motility) was most common, but the majority of cells (>70%) displayed at least three behaviour types. Treatment of sperm with Ca^2+^-mobilising agonists had negligible effects on the rate of switching but increased the time spent in type 2 and type 3 (hyperactivation-like) behaviours (*P* < 2^*^10^−8^; chi-square). Treatment with 4-aminopyridine under alkaline conditions (pH_o_ = 8.5), a highly-potent Ca^2+^-mobilising stimulus, was the most effective in increasing the proportion of type 3 behaviour, biasing switching away from type 1 (*P* < 0.005) and dramatically extending the duration of type 3 events (*P* < 10^−16^). Other stimuli, including 300 nM progesterone and 1% human follicular fluid, had qualitatively similar effects but were less potent. We conclude that human sperm observed *in vitro* constitutively display a range of behaviours and regulation of motility by [Ca^2+^]_i_, at the level of the single cell, is achieved not by causing cells to adopt a ‘new’ behaviour but by changing the relative contributions of those behaviours.

## Introduction

The ability of mammalian sperm to change behaviour is essential, as different types of motility are suited to overcoming the different barriers that the sperm encounters as it ascends the female tract. For instance, sperm from mice that are null for CatSper (the primary Ca^2+^ channel of sperm) fail to hyperactivate and consequently cannot fully ascend the female tract (as they cannot escape after binding the oviduct wall) and are unable to penetrate the zona pellucida (Carlson *et al.*, 2003, Ho *et al.*, 2009). During semen analysis, the motility of a sperm population is typically assessed by estimating quantitative characteristics of the population (% motile, % progressively motile). Use of computer-assisted sperm analysis (CASA) provides more detailed information by tracking the movement of each sperm head, typically for a fraction of a second. With these data, the kinematics of each sperm (usually several hundred cells) are calculated, providing an overview of the nature and variation of motility in the population (Mortimer, 2000). In both cases, the quantitative data are calculated/estimated from a ‘snapshot’ sample, effectively assuming that each cell has a motility type and that the distribution of these types reflects the nature of the population (Pacey *et al.*, 1997).

However, when mammalian (including human) sperm are monitored for longer periods (1–10 s), it becomes apparent that the behaviour of individual cells may change rapidly. For instance, hyperactivated-like motility may occur as intermittent ‘bursts’ interspersed with activated (progressive) swimming (Cooper *et al.*, 1979; Katz and Yanagimachi, 1980; Johnson *et al.*, 1981; Burkman, 1984; Mortimer and Swan, 1995; Pacey *et al.*, 1995). This alternation of behaviour may well be functionally significant, possibly enhancing penetration of the zona pellucida (Bedford, 1998; Katz *et al.*, 1989) or facilitating detachment from the oviduct wall.

Thus, although it is likely that a primary ‘aim’ of stimuli that affect sperm motility is simply to increase the fraction of the population in which a required behaviour occurs, more subtle effects might be achieved at the level of individual cells. For instance, by biasing the probability of switching towards (or away from) a particular behaviour, or by selectively changing the duration of a specific behaviour, the relative amounts of time spent in each behaviour may be regulated. It is also possible that the absolute duration of an individual period of a specific behaviour is functionally important, such as during interaction with the female tract or oocyte vestments.

We hypothesised that behavioural switching and expression of multiple behaviour types is a common feature of human sperm, even under unstimulated conditions. To better understand both the occurrence of multiple patterns of behaviour in individual human sperm and the way in which behavioural switching is regulated, we have (i) recorded and analysed the activity of individual cells over a prolonged period (>3 min) and (ii) investigated whether this behaviour is modified through elevation of [Ca^2+^]_i_.

## Materials and Methods

### Materials

All chemicals were obtained from Sigma-Aldrich (Poole, UK) unless stated otherwise. Progesterone (P4) and 4-aminopyridine (4-AP) were prepared as stocks in dimethylsulphoxide (DMSO) at 10 mM and 500 mM, respectively. Human follicular fluid (FF) was thawed and diluted in saline on the day of use. Thimerosal was prepared as 100 mM stock in deionised water. Working concentrations were made by diluting in supplemented Earle’s balanced salt solution (sEBSS) at the appropriate pH prior to use. DMSO in P4 and 4-AP experiments was 0.003% and 0.4%, respectively. One percent DMSO had no detectable effect on kinematic properties of sperm motility (Achikanu *et al.*, 2018).

### Salines

sEBSS contained (mM) 90 NaCl, 1.017 NaH_2_PO4, 5.4 KCl, 0.81 MgSO4, 5.5 C_6_H_12_O_6_, 2.5 C_3_H_3_NaO_3_, 19 CH_3_CH(OH) COONa, 25 NaHCO_3_, 1.8 CaCl_2_, 25 mM 4-(2-hydroxyethyl)-1-piperazineethanesulfonic acid (HEPES; pH 7.4) and 0.3% BSA. Osmolality was then adjusted to 291–294 mOsm as necessary by adding NaCl. For buffering at pH 8.5, HEPES was replaced with [tris (hydroxymethyl)methylamino] propanesulfonic acid (TAPS).

### Selection and preparation of spermatozoa

Semen samples were from donors with normal sperm concentration and motility (WHO 2010). Samples were obtained by masturbation after 2–3 days sexual abstinence. After liquefaction (30 min), sperm were swum up into sEBSS (60 min), adjusted to ≈6 million/ml and incubated under capacitating conditions (sEBSS at pH 7.4; 37°C, 6% CO_2_) for 5 h (Alasmari *et al.*, 2013a).

### Human FF

FF was prepared as described in Brown *et al.* (2017). Briefly, oocytes (metaphase II) were retrieved by transvaginal aspiration. FF without blood contamination was centrifuged (2500 g for 10 min) to separate cellular components The supernatant (0.22 μm filtered) was stored (at −20°C) until use.

### Ethical approval

Written consent was obtained in accordance with the Human Fertilisation and Embryology Authority Code of Practice (version 8) under local ethical approval (University of Birmingham ERC 07–009 and ERN-12-0570) and (13/ES/0091) from the East of Scotland Research Ethics Service REC1.

### Long-duration sperm tracking

Observation chambers, 20 μm deep, were constructed by mounting a 22 × 32 mm coverslip (0.13–0.16 mm thickness; Academy, Beckenham, UK) onto a 22 × 50 mm coverslip (thickness no. 1.5; VWR International). Soda lime glass beads (18–22 μm; Cospheric, Santa Barbara, USA) were dispersed in vacuum grease (Dow-Corning, USA) and small dabs were placed at each corner of the 22 mm × 32 mm coverslip, providing a ≈ 20 μm space when the two coverslips were pressed together.

Capacitated cells were pelleted by centrifugation and re-suspended in 100 ml aliquots (cell density adjusted to 1.2 million/ml) in sEBSS pH 7.4 or 8.5 as required. The stimulus (1% DMSO control, 300 nM progesterone, 1% FF (containing ≈300 nM P4 (Brown *et al.* 2017)), 1 μM thimerosal or 2 mM 4-AP) was added to the cell suspension and incubation (37°C) was continued for a further 1–2 min. A 5 μl aliquot of cell suspension was carefully loaded into the coverslip chamber, which was then placed on the platform of a thermostatic controller (TC-324B, Warner, USA) set at 36.5°C on the stage of an inverted microscope (Nikon Eclipse TE200) equipped with a motorised stage (Prior Scientific, UK). Images were acquired with a sCMOS camera (Zyla 5.5, ANDOR TM Technology, Belfast, UK) through a 10× phase contrast objective.

Imaging was started ~5 min after stimulus application. Images were acquired at 50 Hz, 15 ms exposure for a period of 180–216 s using Micro Manager software v1.4.22 (image 1392 × 1040 pixels, 16 bit dynamic range, global camera shuttering). Illumination intensity was reduced as far as possible and applied via a red filter to prevent direct effects of illumination on sperm motility (Shahar *et al.*, 2011; Gabel *et al.*, 2018). For each recording, a randomly selected sperm was followed. When the cell approached the edge of the field of view, it was moved back using the motorised stage. Using playback of videos, sperm behaviour was analysed in 1 s blocks, allocating each block to one of four different behaviours (motility types 1, 2, 3 and 4; Table I; Supplementary Fig. S1; see results). Observation of videos gave no indication that stage movements induced or modified behavioural switching (Supplementary Fig. S2). We analysed the relationship between the number of stage movements during a 3 min recording (x) and number of behavioural switches in that 3 min (y). There was no significant relationship (*y* = 0.08x + 18; *P* = 0.9; *R*^2^ = 0.001). Furthermore, in many cells in which switching was frequent, no stage movements were required. We also assessed whether the behaviour changes over the period of the recording might occur if evaporation of saline affected osmolality or pH of the droplet. Neither the rate of switching nor the behaviour score showed a significant trend over the duration of the recording (*P* = 0.83; *P* = 0.58 respectively; Supplementary Fig. S3).

**Table I:** Characteristics of the motility types identified by visual analysis of videos.

Motility type	Characteristics	Progressive/non-progressive	Typical ALH
1	Low amplitude flagellar beat, symmetric or occasionally weakly asymmetric causing curved or circular path	Progressive	≈2–4 μm
2	Intermediate amplitude beat, symmetric or slightly asymmetric (causing circling)	Progressive	≈3–8 μm
3	High-amplitude highly asymmetric beat	Non-progressive, continuous turning or tumbling	≥6 μm
4	Tight bending of the midpiece producing a ‘J’-shape or coil	Non-progressive arrested	n/a

Values shown for typical ALH (amplitude of lateral head displacement) show range of values obtained from examination of 15–20 tracks of each type and are descriptive, not definitive. n/a indicates not applicable.

In a subset of experiments, where the head centroid could be reliably detected throughout the recording, the multidimensional motion analysis application in MetaMorph® (Molecular Devices, USA) was used to generate positional information for the sperm head for the entire recording period. Separate sections of the sperm track (between stage re-centring) were ‘stitched’ together by compensating the offset of ‘x’ and ‘y’ co-ordinates in Microsoft Excel.

### CASA

Hyperactivation was assessed as described previously (Achikanu *et al.*, 2018). After addition of the stimulus, cells were loaded into pre-warmed 20 μm CASA chamber (36°C). Motility was assessed (~300 s after stimulus application) with a Hamilton Thorn CEROS CASA system (version 14.0). Criteria for hyperactivation were VCL (curvilinear velocity) ≥150 μm/s, LIN (linearity) < 50% and ALH (amplitude of lateral head movement) ≥7 μm (Mortimer, 2000).

### Data analysis

Fractal dimension (FD) was determined from x, y coordinates over a period of 1 s (50 points) for each track point along the trajectory. FD was calculated using the equation

(1) \begin{equation*} FD = log(n)/[log(n) + log\ (ld/cd)]\nonumber\end{equation*}

where n is the number of points in the interval analysed (50 points), ld is the linear distance between the first and the last point and cd is the length of the trajectory (Mortimer *et al.*, 1996). FD was calculated with a homemade macro (paNoel 1.0; Universidad Nacional de Córdoba; http://www.iibyt.conicet.unc.edu.ar/software/) using Fiji software (Schindelin *et al.*, 2012). Maximum FD was set at 2.0.

Behaviour score (average of all the recorded scores for each 1 s period over the entire recording), % time (% of total recording period spent in each behaviour type), switching rate (transitions.min^−1^), behaviour dwell time (period during which a single behaviour type occurred) and transition type (behaviour type entered upon a transition) were calculated in Microsoft Excel and statistical analysis was carried out using Minitab18 and Excel. Behaviour score, % time and switching rate were calculated separately for each cell and a mean was calculated from these values (*n* = number of cells analysed). Dwell times and transition types were analysed using all individual behaviour events of the specified type (*n* = total number of events). All summary values given in the text are mean ± SEM unless stated otherwise. Before analysis, data were tested for normality (Anderson–Darling) and data sets were then compared using chi-square, Student’s *t*-test, Mann–Whitney, 2-way analysis of variance (ANOVA) and Kruskal–Wallis (with *post-hoc* Holm–Bonferroni sequential correction; Gaetano, 2013) as appropriate. Autocorrelation assessment of periodicity was carried out in Minitab 18.

## Results

Using five conditions (control and four different stimuli), each at two different values of pH_o_ (7.4 and 8.5), we collected video recordings from a total of 180 cells. By visual analysis of video playback, we categorised behaviour into four types. Types 1, 2 and 3 resembled the patterns of motility normally referred to as activated, transitional and hyperactivated (Supplementary Fig. S1, Table I; Supplementary Videos 1 and 2; Mortimer, 2000). An additional type of behaviour was occasionally observed, in which the mid-piece and anterior flagellum became tightly curved (forming a ‘J’ shape or coil) and arrested or ‘twitched’ (Supplementary Video 3, Table I). Since behaviour of this type is seen in cells treated with Ca^2+^-ionophore, where it apparently reflects excess Ca^2+^-loading that takes the cells ‘beyond’ hyperactivated motility (Sanchez-Cardenas *et al.*, 2018), this was designated type 4 behaviour. This behaviour was typically interspersed with very brief (100–500 ms) bursts of flagellar activity such that the cell became virtually immobilised for up to 20 s (Supplementary Fig. S1d, Supplementary Video 3).

**Figure 1:**
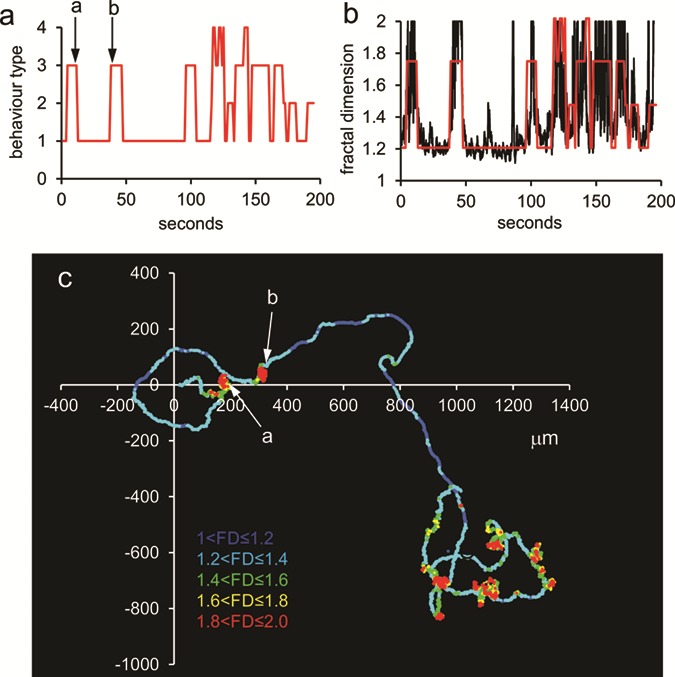
**Behavioural switching in a free-swimming human sperm under control conditions (pH**_**o**_ **= 7.4).** (**a**) Variation in behaviour type (categorised visually as type1, type 2, type 3 or type 4; see Supplementary Figure S1) of a single sperm over a period of 190 s. (**b**) Variation in fractal dimension (FD) over time (black trace) overlaid with the visually categorised behaviour types (red trace). Visual analysis and FD show good agreement, with no visually identified behavioural transitions that are not confirmed by FD. (**c**) Track of the same cell, colour coded to display variation in the fractal dimension (FD); (1 < FD ≤ 1.2 (dark blue); 1.2 < FD ≤ 1.4 (light blue); 1.4 < FD ≤ 1.6 (green); 1.6 < FD ≤ 1.8 (yellow); 1.8 < FD ≤ 2.0 (red)). Axes show distance in μm.

### Free-swimming sperm cells repeatedly ‘switch’ behaviour

**Figure 2:**
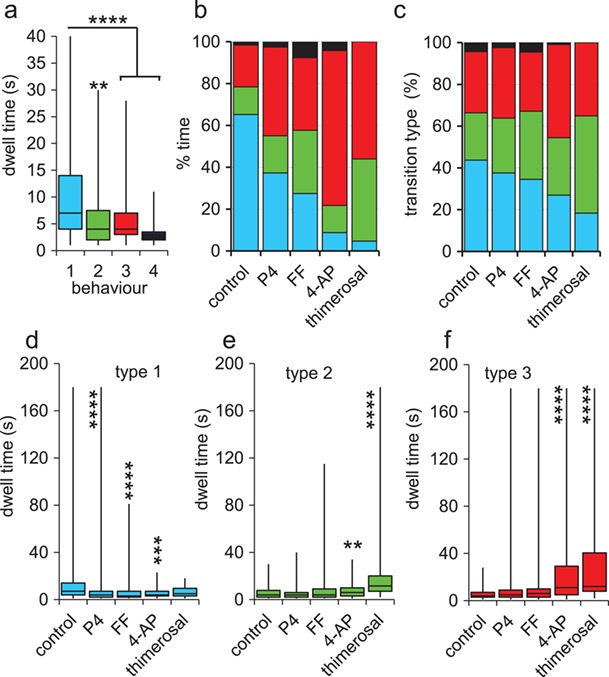
**Characteristics of behavioural switching.** (**a**) Dwell times (period during which a single behaviour type occurred) for types 1 (light blue, *n* = 164 events), 2 (green, *n* = 79), 3 (red, *n* = 107) and 4 (black, *n* = 15) behaviours in cells control cells (pH 7.4). Plots show median and interquartile range (box) and maximum/minimum values (whiskers). (**b**) Mean % time spent in each behaviour; type 1 (light blue), type 2 (green), type 3 (red) and type 4 (black) under control conditions (*n* = 18 cells) and in the presence of 300 nM P4 (*n* = 18 cells), 1% FF (*n* = 17 cells), 2 mM 4-AP (*n* = 21 cells) and 1 μM thimerosal (*n* = 17 cells). (**c**) Relative frequencies of transitions into type 1 (light blue), type 2 (green), type 3 (red) and type 4 (black) behaviours under control conditions (*n* = 347 transitions) and in the presence of progesterone (P4; 300 nM; *n* = 420), human follicular fluid (FF nM, 1%; *n* = 353), 4-aminopyridine (4-AP, 2 mM, *n* = 211) and thimerosal (1 μM, *n* = 115). Asterisks indicate significant difference from control; ^***^ = *P* < 0.005. (**d–f**) Dwell times for type 1 (panel ‘d’; blue), type 2 (panel ‘e’; green) and type 3 (panel ‘f’; red) behaviours under control conditions and in the presence of 300 nM progesterone (P4), 1% human follicular fluid (FF), 2 mM 4-aminopyridine (4-AP) and 1 μM thimerosal. Plots show median and interquartile range (box) and maximum/minimum values (whiskers) of 51–167 events. Asterisks indicate significant difference from control; ^**^ = *P* < 0.01; ^***^ = *P* < 0.001; ^****^ = *P* < 0.0001.

Examination of the behaviour of cells incubated under control conditions (sEBSS; pH 7.4) showed that the majority (16/18 cells) changed their behaviour at least once during the recording. Type 1 behaviour was seen in 18/18 cells, type 3 occurred in 16/18 (no significant difference in incidence; *P* = 0.15; chi-square) and 13 of these cells showed three or (in one case, Fig. 1a) all four types of behaviour. Among the 18 control cells analysed, the number of behavioural transitions varied widely (from 0 to 45), with the mean rate being 6.4 ± 0.8 transitions.min^−1^. Dwell-times (length of period during which a single behaviour type occurred) for types 1, 2 and 3 events all formed negative exponential distributions (Supplementary Fig. S4a), with the median duration of type 1 events being significantly greater than those for types 2, 3 and 4 (*P* < 5^*^10^−5^; Kruskal–Wallis; Fig. 2a). Analysis of transition types showed that switching between behaviours was not random (*P* < 10^−10^; chi-square), with transitions into type 4 behaviour being very rare (4.3%) and into type 1 behaviour occurring most frequently (44%; Fig. 2c column 1). Consequently, cells spent most time (65 ± 6%) in type 1 behaviour, more than double that of any of the other behaviour types (Fig. 2b column 1; *P* < 0.0005; Kruskal–Wallis with *post-hoc* comparison). Occurrence of type 4 behaviour was very rare, making up <3% of behaviour events and of total time (Fig. 2b). Neither the rate of switching nor the type of behaviour expressed showed a significant trend over the recording period (*P* = 0.83; *P* = 0.58 respectively; Supplementary Fig. S3).

In most cells where behavioural switching occurred, there was no clear pattern, as type 1 behaviour was interrupted by ‘bursts’ of type 2 and type 3 activity (Fig. 1a). In a small number of cells (4/18), behaviour appeared to oscillate but analysis by autocorrelation showed only one instance where an oscillatory frequency emerged (Supplementary Fig. S5, compare to Fig. 1) and even in this case, the autocorrelation failed to reach significance (*P* > 0.05; Supplementary Fig. S5b). For 3 of the 18 control cells, where video quality was adequate for reliable detection of the sperm head throughout the 3 min recording, we were able to reconstruct entire tracks lasting over 3 min, which clearly showed the observed transitions in behaviour (Fig. 1c). To confirm that visual analysis identified genuine behavioural transitions, we used the track coordinates to assess temporal variation of FD (a numerical value between 1 and 2, which reflects the complexity of the sperm track; Mortimer *et al.*, 1996). Colour coding of track points for FD confirmed that high values occur at periods of complex motility and overlays of FD and visual analyses confirmed that the same behavioural transitions were identified (Fig. 1b and c, Supplementary Fig. S5c and d, Supplementary Fig. S6). Autocorrelation of the FD data in Fig. S5 produced near identical results to those obtained through visual analysis (Supplementary Fig. S5b and e). When data from all tracked cells were analysed, the mean FD and mean behavioural score (average score over the entire recording) for each cell were clearly correlated (*R*^2^ = 0.56; *P* = 5^*^10^−6^; *n* = 28; Supplementary Fig. S7).

### Manipulation of [Ca^2+^]_i_ modifies the pattern of behavioural switching

A pivotal mechanism for regulation of behaviour in human sperm is [Ca^2+^]_i_ (Publicover *et al.*, 2007; Suarez, 2008). To investigate the effect of [Ca^2+^]_i_ elevation, we monitored the activity of cells exposed to four different stimuli (300 nM P4, 1% FF [containing ~300 nM P4], 2 mM 4-AP and 1 μM thimerosal), all of which are known to increase [Ca^2+^]_i_ and stimulate hyperactivation in human and other sperm, but with varying potency (Ho and Suarez, 2001; Alasmari *et al.*, 2013b; Brown *et al.*, 2017; Achikanu *et al.*, 2018). CASA assessments, carried out during the period of behavioural data collection, using cells prepared under identical conditions, confirmed the efficacy and relative potency of these stimuli (Supplementary Fig. S8a). The proportion of cells in which we observed switching between behaviours was not changed by any of these manipulations (Table II), but the relative incidence (% time) of the different behaviour types was significantly modified (*P* < 2^*^10^−8^; chi-square). Incidence of type 1 behaviour was reduced by all four stimuli (*P* < 0.05), whereas 4-AP significantly increased the incidence of type 3 behaviour (*P* = 2^*^10^−6^) and thimerosal significantly increased the incidence of both types 2 and 3 behaviours (*P* < 0.05; Kruskal–Wallis with *post-hoc* comparison; Fig. 2b). Examination of switching showed that this change was partly due to biasing of switching towards types 2 and 3 behaviours in stimulated cells (P4 *P* = 0.1; FF *P* < 0.05; 4-AP and thimerosal *P* < 5^*^10^−5^; chi-square; Fig. 2c), but there were also marked effects on dwell times of the different behaviours. Type 1 events were significantly briefer in stimulated cells (except for those exposed to thimerosal; Fig. 2d), whereas type 2 and type 3 events were markedly prolonged in cells exposed to 4-AP and to thimerosal (Fig. 2e and f; Supplementary Fig. S6), such that the overall frequency of behaviour switching was significantly lower than in controls (*P* = 0.02 and *P* = 0.0004 for 4-AP and thimerosal respectively; Kruskal–Wallis).

**Table II:** Incidence of behavioural switching.

	Treatment				
pH_o_	Control	P4	FF	4-AP	Thimerosal
7.4	16/18	16/18	16/17	20/21	14/19
8.5	16/18	13/15	14/18	10/20	11/16
P	1	1	0.67	0.01	1

Data show proportion of cells in which behavioural switching was observed (number of cells showing switching/total number of cells analysed) at pH_o_ = 7.4 and pH_o_ = 8.5 under control conditions and in the presence of 300 nM progesterone (P4), 1% human follicular fluid (FF), 2 mM 4-aminopyridine (4-AP) and 1 μM thimerosal. The bottom row of the table (P) shows results of chi-square test to compare between pH values (corrected for multiple comparisons) under each condition.

### Effect on behaviour of elevated pH_o_

**Figure 3:**
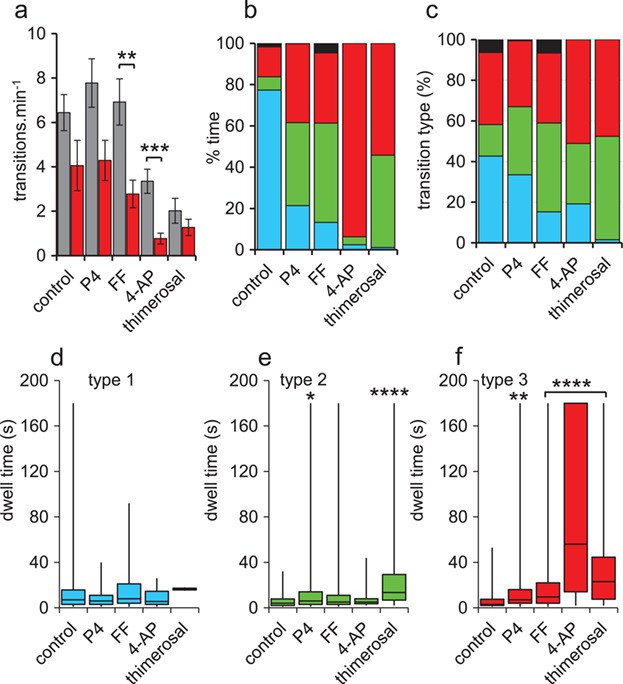
**Characteristics of behavioural switching at pH**_**o**_ **= 8.5.** (**a**) Mean switching rate (±s.e.m.) for cells incubated at pH_o_ = 7.4 (grey bars) and pH_o_ = 8.5 (red bars) under control conditions (*n* = 18 cells) and in the presence of 300 nM P4 (*n* = 18 cells), 1% FF (*n* = 17 cells), 2 mM 4-AP (*n* = 21 cells) and 1 μM thimerosal (*n* = 17 cells). (**b**) Mean % time spent in each behaviour at pH_o_ = 8.5; type 1 (light blue), type 2 (green), type 3 (red) and type 4 (black) under control conditions (*n* = 18 cells) and in the presence of 300 nM P4 (*n* = 15 cells), 1% FF (*n* = 18 cells), 2 mM 4-AP (*n* = 20 cells) and 1 μM thimerosal (*n* = 16 cells). (**c**) Relative frequencies of transitions into type 1 (light blue), type 2 (green), type 3 (red) and type 4 (black) behaviours at pH_o_ = 8.5 under control conditions (*n* = 220 transitions) and in the presence of progesterone (P4; 300 nM; *n* = 194), human follicular fluid (FF nM, 1%; *n* = 151), 4-aminopyridine (4-AP, 2 mM, *n* = 47) and thimerosal (1 μM, *n* = 63). (**d–f**) Dwell times at pH_o_ = 8.5 for type 1 (panel ‘d’; blue), type 2 (panel ‘e’; green) and type 3 (panel ‘f’; red) behaviours under control conditions and in the presence of 300 nM progesterone (P4), 1% human follicular fluid (FF), 2 mM 4-aminopyridine (4-AP) and 1 μM thimerosal. Plots show median and interquartile range (box) and maximum/minimum values (whiskers) of 10–110 events (except thimerosal type 1 where *n* = 2). Asterisks indicate significant difference from control; ^*^ = *P* < 0.05; ^**^ = *P* < 0.01; ^***^ = *P* < 0.001; ^****^ = *P* < 0.0001.

To investigate the effect on behavioural switching of elevated pH_o_, sperm were prepared using the standard protocol but then re-suspended in saline buffered at pH 8.5, which causes stable alkalinisation of the cytoplasm (increase from 6.9 to 7.2) within 200–300 s and increases levels of hyperactivated motility (Achikanu *et al.*, 2018; Supplementary Fig. S8a). The proportion of sperm in which switching occurred was not significantly changed at elevated pH except in cells exposed to 4-AP, where switching was observed in only 10/20 cells (*P* = 0.005 compared to 4-AP at pH 7.4; Table II). The frequency of switching at pH_o_ = 8.5, compared to cells incubated at pH_o_ = 7.4, was consistently lower (*P* < 2^*^10^−7^, 2-way ANOVA; Fig. 3a), but when the five conditions were analysed separately, this effect of pH was significant only in cell exposed to FF (*P* < 0.01) and 4-AP (*P* < 0.001; Mann–Whitney). Similarly to our observations at pH_o_ = 7.4, at pH_o_ = 8.5 all of the stimuli significantly changed the relative abundance (% time) of the four behaviours (Fig. 3b; *P* < 10^−14^; chi-square), with the most striking effect being in 4-AP-treated cells, where sperm spent 94 ± 2% of their time in type 3 behaviour (Fig. 3b; *P* = 3^*^10^−9^ compared to control; Kruskal–Wallis with *post-hoc* comparison). Examination of the effect of stimuli on the characteristics of transitions between behaviours also showed that, as at pH 7.4, Ca^2+^-mobilising treatments biased switching events towards types 2 and 3 behaviour (Fig. 3c; *P* < 0.005; chi-square). Dwell times for type 1 behaviour were not significantly altered by Ca^2+^-mobilising stimuli at pH 8.5, but the effects of agonists on type 3 dwell times were more consistent and greater than at pH_o_ = 7.4, with all four stimuli significantly extending the duration of type 3 behaviour compared to control cells (Fig. 3f; *P* < 0.001; Kruskal–Wallis with *post-hoc* comparison). Most strikingly, in the presence of 4-AP, type 3 behaviour median dwell time was 56 s (compared to 3 s in controls; *P* = 10^−16^, Kruskal–Wallis with *post-hoc* comparison; Fig. 3f). Only thimerosal significantly increased type 2 dwell times at both pH_o_ = 7.4 and pH_o_ = 8.5 (*P* = 10^−10^ and *P* = 10^−5^, respectively; Kruskal–Wallis with *post-hoc* comparison). Comparison of mean behaviour scores and % hyperactivated cells (CASA) for the 10 conditions assessed (control and four agonists, each at pH 7.4 and pH 8.5) showed a positive correlation between the two assessments of motility (*R*^2^ = 0.5; *P* = 0.022; Supplementary Fig. S8b).

## Discussion

By continuously observing ‘capacitated’, free-swimming human sperm, for a period of longer than 3 min, we have shown, for the first time, the occurrence of continuous switching between different types of motility, with the mean incidence being more than six transitions per minute. Even in control (unstimulated) cells, multiple behaviours, including hyperactivated-like motility, were seen in ≈90% of cells. For analysis of this switching, the intervening periods, where behaviour appeared consistent, were classified using four categories. Although tracks from behaviour types 1–3 resemble those referred to as activated, transitional and hyperactivated motility (Mortimer, 2000), these behaviours were derived by visual analysis and cannot be considered equivalent to categories defined by kinematic criteria. Type 4 behaviour where the flagellum arrested, typically in a ‘J’ shape, appears to be equivalent to the freeze and freeze-flex behaviours, which were observed in ≈7% of cells from normal semen (Burkman, 1984). In cells where analysis of FD was possible, it was clear that (i) visual analysis and FD identified the same behavioural transitions and (ii) for each recording, the mean FD was clearly and positively correlated with mean behaviour score (*R*^2^ = 0.56). We conclude that our analysis reliably identifies sperm behaviours and switching between them and that continuous behavioural switching is a normal activity of human sperm (at least for cells observed *in vitro*). Though some behaviour-time plots appeared to show oscillatory behaviour, autocorrelation analysis did not reveal significant periodicity.

The occurrence of repeated behavioural switching might be brought about by either, or both, of two different mechanisms. Changes in behaviour might be completely reliant on endogenous signalling activity, for instance, [Ca^2+^]_i_ spikes and oscillations that have been described in human sperm (Harper *et al.*, 2004; Aitken & McLaughlin, 2007; Sanchez-Cardenas *et al.*, 2014; Mata-Martinez *et al.*, 2018). These changes were observed in immobilised cells and were correlated with changes in flagellar beating (Harper *et al.*, 2004; Bedu-Addo *et al.*, 2007). However, it is yet to be established whether these [Ca^2+^]_i_ signals cause or are caused by the changes in flagellar beating. Alternatively, if mechanisms controlling sperm behaviour are sensitive to changes in the environment, such as pH, ionic environment, temperature and mechanical stimulation (including fluid flow), heterogeneity in the sperm’s environment might present such ‘stimuli’ as it swims. Our data indicate that potential methodological artefacts related to movements of the stage and/or evaporation of the saline did not cause such effects (see Methods; Long Duration Sperm Tracking). However, the environment through which the sperm swims, even in the simple system used for our recordings, might be sufficiently spatially heterogeneous that the sperm encounters a series of physico-chemical ‘stimuli’ due to its own movement. The complex environment encountered by the sperm in the female tract will almost certainly present such ‘stimuli’. If behavioural switching is induced in this way, our observation that [Ca^2+^]_i_ elevating stimuli prolong periods of type 3 behaviour (Figs 2f and 3f; see below) might indicate that Ca^2+^-signalling resets the stimulus sensitivity of transition into this type of motility. In either case, it appears likely that conditions/stimuli that result in behavioural switching *in vitro* will also occur *in vivo*.

Analysis of the effects of manoeuvres that elevate [Ca^2+^]_i_ in human sperm indicate that motility kinematics (assessed by CASA) are directly related to the absolute level of [Ca^2+^]_i_, irrespective of the Ca^2+^ source mobilised, with the percentage of hyperactivated cells increasing as a function of [Ca^2+^]_i_ (Achikanu *et al.*, 2018). Exposure of cells to a range of stimuli that elevate [Ca^2+^]_i_ significantly altered the relative abundance of the different behaviours. Consistent with a shift towards hyperactivated motility, the proportion of time spent in type 2 and type 3 behaviours markedly increased in cells exposed to Ca^2+^-mobilising stimuli. Thimerosal and 4-AP were particularly potent in this regard. Thimerosal modifies sperm behaviour by mobilising stored Ca^2+^ (Ho and Suarez, 2001; Alasmari *et al.*, 2013b). Although 4-AP is often used as a K^+^ channel blocker, its effects on the K^+^-permeable channels expressed in sperm are negligible (Tang *et al.*, 2010; Mansell *et al.*, 2014) and a significant aspect of its action on sperm [Ca^2+^]_i_ and behaviour is likely to be its ability to mobilise stored Ca^2+^ (Gobet *et al.*, 1995; Grimaldi *et al.*, 2001; Bhaskar *et al.*, 2008;^4,22^). The potent effects of Ca^2+^-store-mobilising stimuli on switching are consistent with their ability to persistently elevate [Ca^2+^]_i_, inducing prolonged hyperactivation in human sperm (Achikanu *et al.*, 2018; Alasmari *et al.*, 2013a,b). When cells were suspended in medium buffered at pH 8.5, some effects of Ca^2+^-mobilising stimuli were greatly enhanced, particularly the prolongation of type 3 behaviour dwell-time by 4-AP. The effect of 4-AP on [Ca^2+^]_i_ is strongly potentiated at pH = 8.5 (Achikanu *et al.*, 2018) and it is likely that this underlies the striking effect of the drug on behavioural switching under these conditions. Significantly, these potent hyperactivating stimuli had negligible effect on the proportion of cells in which type 3 (hyperactivated-like) behaviour was observed. Even under control conditions, type 3 behaviour (and behavioural switching) occurred in 90% of cells. Thus, it appears that the well-characterised ability of these [Ca^2+^]_i_-mobilising compounds to increase the level of hyperactivation detected in population motility assays occurs not by recruitment of cells into a hyperactivated population, but by increasing the proportion of time that continuously-switching cells spend in type 3 (hyperactivated-like) motility, achieved primarily by extending the dwell-time of this behaviour.

If behavioural switching occurs *in vivo*, does it have adaptive value? Switching induced externally (by the sperm’s sensitivity to heterogeneity in its environment; see discussion above) may simply be behavioural ‘noise’ that can be modulated by the cells signalling activity (such as elevation of [Ca^2+^]_i_ as described here) but may not be functionally significant. However, observations of sperm interacting with the female tract and cumulus-oocyte-complex suggest that switching of motility types may be of value. Pacey *et al.* (1995) described an attach–detach cycle in the interaction of human sperm with epithelial cells isolated from the isthmic and ampullary sections of human oviducts, which may play an important role in migration to the fertilisation site. Detachment was associated with hyperactivated-type motility, whereas cells that were attaching showed far more linear motility. Similarly, observation of sperm during the passage through the zona reveals alternation of low and high amplitude flagellar beats, which may facilitate penetration by alternation of ‘cutting’ and ‘thrusting’ forces (Bedford, 1998). Clearly further work is required to determine the nature and complexity of sperm behaviour *in vivo* and its significance.

## Authors’ roles

C.A., J.C. and S.M.D.S. obtained samples and carried out the laboratory work. C.A., S.P. and H.G. analysed the data. C.A., J.C., H.G., L.G., C.L.R.B., S.M.D.S. and S.J.P. contributed to the writing of the manuscript.

## Funding

Medical Research Council (MR/M012492/1); C.A. was in receipt of a scholarship from the Nigerian government (Tertiary Education Trust (TET) Fund).

## Conflict of interest

C.L.R.B. was the editor-in-chief of Molecular Human Reproduction, has received lecturing fees from Merck and Ferring and is on the Scientific Advisory Panel for Ohana BioSciences. C.L.R.B. was also the chair of the World Health Organization Expert Synthesis Group on Diagnosis of Male infertility (2012–2016). He is an editor of RBMO. C.L.R.B. reports grants from the MRC, Gates Foundation and the Scottish Office during the conduct of the study; personal fees from Pharmasure, Ferring, Ohana and RBMO outside the submitted work. C.A., J.C., H.G., L.G., S.M.D.S. and S.J.P. declare no conflict of interest.

## Supplementary Material

Supplementary_data_gaz034
