# Supplementary material for: Continuous behavioural ‘switching’ in human spermatozoa and its regulation by Ca2+-mobilising stimuli
Source: Mol Hum Reprod. 2019 Jun 13;25(8):423–32. doi: 10.1093/molehr/gaz034 (PMC6736438; doi:10.1093/molehr/gaz034)
Supplement: Supplementary_data_gaz034 [file supplementary_data_gaz034.zip › MHR-19-0058-R1-SuppFigs GAZ034.docx]

Figure S1. Sperm behaviour types. Panels a-d show examples of type 1, type 2, type 3 and type 4 behaviours: (a) type 1, 1.88 s track; (b) type 2, 2.9s s track; (c), type 3, 11.2 s track; (d) type 4, 5 s track. Black lines and points show sperm track and position of sperm head centroid in each video frame (interval=20 ms). Red lines show 15 point average path. Blue arrows indicate start of track. Grid lines show 20 μm (major) and 2 μm (minor). Note that in panel ‘d’ two groups of points indicating position of the head centroid are tightly clustered due to near arrest of flagellar movement.

Fig S2. Stage movements do not induce behavioural switching. Four examples of cells repeatedly switching between behaviour types (see Figure S1). Times at which stage movements were made are marked by vertical red bars. Incubation conditions were; (a) pH_o_=7.4 with 300 nM P4; (b) pH_o_=7.4 with 0.1% FF; (c) pH_o_=8.5 with 2 mM 4-AP; (d) pH_o_=8.5 with 1 μM thimerosal.

Figure S3. Absence of temporal trends in behaviour and switching rate. (a) Mean behaviour score over 3 min of recording for cells incubated under control conditions at pH_o_=7.4 (black; n=18 cells) and in the presence of 300 nM progesterone at pH_o_=7.4 (P4; blue; n=18) and 2 mM 4-AP at pH_o_=8.5 (red; n=20), the most potent [Ca^2+^]_i_ stimulus tested. (b) Mean regression coefficients (±s.e.m; expressed as Δ behaviour score.s^-1^) for the data plotted in panel ‘a’, obtained by calculating the individual linear regressions for each cell. None of these means differ significantly from zero. (c) Variation of behavioural switching rate (transitions.min^-1^) over 3 min of recording. Data were assessed in 20 s bins. Black shows cells incubated under control (n=18 cells), blue shows cells incubated in the presence of 300 nM P4 (n=18) and red shows cells incubated in the presence of 2 mM 4-AP at pH_o_=8.5 (n=20). Points show mean ± SEM. Fitted lines are linear regressions. P values for regression coefficients are 0.83, 0.33 and 0.45 for control (pH_o_=7.4), P4 (pH_o_=7.4) and 4-AP (pH_o_=8.5) respectively.

Figure S4. Distribution of behaviour dwell times in the absence and presence of Ca^2+^-mobilising stimuli. Each panel shows the frequency distribution for dwell times of type 1 behaviour (blue), type 2 (green) and type 3 (red) expressed as % of total frequency. Cells were: (a) incubated under control conditions (n=79-164 events); (b) treated with 300 nM progesterone (P4, n=112-167 events); (c) treated with 1% human follicular fluid (FF, n=105-128 events); (d) treated with 2 mM 4-aminopyridine (4-AP; n=60-108 events); or (e) treated with1 μM thimerosal (n=23-60 events). Note that the x-axis (time in seconds) is logarithmic.

Figure S5. Rhythmic behavioural switching in a free-swimming human sperm under control conditions. (a) Variation in behaviour type (categorised visually as type1, type 2, type 3 or type 4; see Figure S1) of a single sperm over a period of 188 s. (b) Autocorrelation plot for the data shown in panel ‘a’. (c) Track of the same cell, colour coded to display variation in the fractal dimension (FD); 1<FD≤1.2 (dark blue); 1.2<FD≤1.4 (light blue); 1.4<FD≤1.6 (green); 1.6<FD≤1.8 (yellow); 1.8<FD≤2.0 (red). Axes show distance in μm. (d) Variation in fractal dimension over time (black trace) overlaid with the visually categorised behaviour types (red trace). Visual analysis and FD show good agreement, with no visually identified behavioural transitions that are not confirmed by FD. (e) Autocorrelation plot for the FD data (black trace) shown in panel ‘d’.

Figure S6. Behavioural switching in a free-swimming human sperm in the presence of 2 mM 4-aminopyridine at pH_o_=7.4. (a) 215 s track colour coded to display variation in the fractal dimension (FD); 1<FD≤1.2 (dark blue); 1.2<FD≤1.4 (light blue); 1.4<FD≤1.6 (green); 1.6<FD≤1.8 (yellow); 1.8<FD≤2.0 (red). Axes show distance in μm. (b) Variation in behaviour type of the same cell (categorised visually as type1, type 2, type 3 or type 4; see Figure S1). (c) Variation in fractal dimension over time (black trace) overlaid with the visually categorised behaviour types (red trace). Visual analysis and FD show good agreement, with no visually identified behavioural transitions that are not confirmed by FD.

Figure S7. Behavioural score is correlated with fractal dimension. Plot shows relationship between mean fractal dimension and mean behaviour score (both calculated over the entire recording) from cells where both analyses were available. Data from 28 recordings are shown, including cells incubated under control and stimulated conditions. Data show a clear linear relationship (y=0.28x+0.95; R^2^=0.56).

Fig. S8. Ca^2+^-elevating stimuli have similar effects on CASA-assessed hyperactivation and on behaviour score. (a) % hyperactivated cells for control conditions and in the presence of the four Ca^2+^-mobilising stimuli at pH_o_=7.4 (grey bars) and pH_o_=8.5 (red bars). Each bar shows mean ± SEM of 11 determinations, each made on a different day; *=P<0.05; **=P<0.01; ***=P<0.001 compared to control at same pH_o_. (b) Relationship between mean behaviour score for each of the ten conditions assessed (control and four agonists, each at pH 7.4 and pH 8.5; n=15-21 cells) and the mean CASA assessment (n=11) of % hyperactivated cells under the same conditions (P value for regression coefficient=0.022).
