# Supplementary material for: Continuous behavioural ‘switching’ in human spermatozoa and its regulation by Ca2+-mobilising stimuli
Source: Mol Hum Reprod. 2019 Jun 13;25(8):423–32. doi: 10.1093/molehr/gaz034 (PMC6736438; doi:10.1093/molehr/gaz034)
Supplement: Supplementary_data_gaz034 [file supplementary_data_gaz034.zip › Video_legends-revised GAZ034.pdf]

Video Legends

Video 1. This cell repeatedly switches behaviour. Type 1 behaviour occurs at the start of the video and also for the periods 3-5.5 s and 17.5-20 s as shown by the time stamp (seconds) at the top left of the image. Periods of type 3 behaviour occur at approximately 2-3 s, 10-11.5 s and 14-17.5 s. Between 5.5 and 10 s both type 3 and (briefly) type 2 behaviours occur. Total duration= 20 s. Frame dimensions = 230*225 μm

Video 2. Cell showing type 2 behaviour. Total duration= 6 s. Frame dimensions = 230*240 μm

Video 3. Cell showing type 4 (arrested) motility, where the flagellum arrests in a J shape, interspersed by brief periods (< 1 s) of flagellar beating. Total duration= 5.9 s. Frame dimensions = 205*180 μm
